# Is There Any Alternative to Canonical DNA Barcoding of Multicellular Eukaryotic Species? A Case for the Tubulin Gene Family

**DOI:** 10.3390/ijms18040827

**Published:** 2017-04-13

**Authors:** Diego Breviario

**Affiliations:** Istituto Biologia e Biotecnologia Agraria, Consiglio Nazionale delle Ricerche, 20133 Milano, Italy; breviario@ibba.cnr.it; Tel.: +39-022-3699441

**Keywords:** DNA barcoding, molecular taxonomy, tubulin-based polymorphism, species identification, biodiversity

## Abstract

Modern taxonomy is largely relying on DNA barcoding, a nucleotide sequence-based approach that provides automated species identification using short orthologous DNA regions, mainly of organellar origin when applied to multicellular Eukaryotic species. Target DNA loci have been selected that contain a minimal amount of nucleotide sequence variation within species while diverging among species. This strategy is quite effective for the identification of vertebrates and other animal lineages but poses a problem in plants where different combinations of two or three loci are constantly used. Even so, species discrimination in such plant categories as ornamentals and herbals remain problematic as well as the confident identification of subspecies, ecotypes, and closely related or recently evolved species. All these limitations may be successfully solved by the application of a different strategy, based on the use of a multi-locus, ubiquitous, nuclear marker, that is tubulin. In fact, the tubulin-based polymorphism method can release specific genomic profiles to any plant species independently from its taxonomic group. This offers the rare possibility of an effective yet generic genomic fingerprint. In a more general context, the issue is raised about the possibility that approaches alternative to systematic DNA sequencing may still provide useful and simple solutions.

We are leaving at the eve of major social changes, and great are the expectations on the contributions that will be provided by artificial intelligence, largely developed and operating on vast collections of data. Science and technology have entered a new paradigmatic era, where traditional hypothesis-driven experiments are being replaced by data mining, statistical analysis, and predictive modeling [1]. Accordingly, the role of scientists is changing. They must master computing sciences and critically supervise the outputs. In the near future, the majority of life science scientists will be mainly working in offices supported by large computer facilities and working much less in laboratories performing experiments. Why bother to set up experiments and verify hypotheses when you can send out the work to few, dedicated facilities and receive back a substantial amount of information that you can then conveniently process in your office? The question is: Are we sure that this new paradigm will eventually explain the marvelous complexity of biology? The elaborate yet precise network that governs in time and space the growth and development of the living organisms? Take tubulin, for instance. As far as we know, it is per se a very annoying structurally limited protein with just one task to be accomplished: to build up microtubules. Genes encoding tubulin are very easy to annotate in any of the dozens of genome sequencing programs currently run. Even so? annotation, e.g., does not explain the multi-tubulin hypothesis, i.e., why multiple genes for the same, apparently dull function are required, nor does it explain the role of each of the many different post-translational modifications to which tubulin is subjected, the consequences they may have on microtubule interactions and dynamics, how the regulatory role of tubulin introns is exerted, nor even why they are largely conserved in the same positions once established in a lineage of higher EUK evolution [2]. Thus, a different way of efficiently barcoding plant species is to be explored. An exception to the rules.

Today in science, when speaking about DNA barcoding, everybody correctly refers to the CBOL (http://www.barcodeoflife.org), committed, for several years, to identifying suitable markers and collecting data in support of the new taxonomy based on molecular evidence. A DNA barcode is thus indicated in short sequences (400–800 bp) that, routinely amplified and deciphered, are capable of discriminating all living species: microbes, fungi, animals, and plants [3]. To this purpose, certain specific short regions of DNA, often of organellar origin, subjected to a convenient rate of mutation, have been adopted as the most useful barcoders in higher Eukaroytes. In animal species, DNA barcoding is essentially based on the use of a portion of the mitochondrial *cox1* gene, while in plants the situation is trickier because one cannot quite rely on a single sequence of reference, and the concomitant use of more target genes, almost exclusively of chloroplast origin, is often not conclusive [4]. In addition, problems of species classification and attribution remain unsolved in several important cases, such as those of herbals and ornamental plants. Scientists have long been looking for a possible universal, standard DNA barcode for plants without success. They seem to have accepted that multiple markers is the only way to obtain an acceptable level of species discrimination. Inherently they must also accept that handling, managing, and processing barcoding data in plants require heavier informatic support than those in animals. Recently, ITS2 has been suggested as a possible candidate, but conclusive evidence should be further presented [5]. Even so, a major limitation of classical DNA barcoding is dependent on the ability of recognizing intraspecific from interspecific genetic variation [6]. In fact, species discrimination is currently done by accepting a certain degree of sequence divergence that may occur among individuals and within varieties of the same species. This may be difficult when assessing the identity of closely related and newly emerging species compared to different varieties of the same species, eventually resulting in the absence of a clear barcoding gap. These comprehensible adjustments make the use of the word “barcoding” a little loose, strictly speaking, because the sequence information is actually condensed in minimal binnings. It could not be any different because classical species barcoding is based on nucleotide sequencing that can show minimal variation and is prone to errors. Of course, given the premise, fine species discrimination will eventually take further advantage by massive sequencing but the question is: Are we sure that, when applied to the simple purpose of species recognition, there is not something else, some alternative yet comparable analytical approach that can provide a similar information in an easier and more straightforward way? The case is raised in favor of Tubulin-Based Barcoding (TBB), hitherto reported in the literature as tubulin-based polymorphism (TBP).

TBB (ex-TBP) was developed on deep thinking and seems to be as effective, if not more effective, in species recognition than does sequencing. TBB efficacy ultimately depends on the biological role of tubulin, one of the, if not the most conserved protein in evolution. In fact, Eukaryotes have evolved for thousand of millions of years while preserving the same mechanism of cell division, almost exclusively based on microtubules and their ability to recruit chromosomes and favor their movements to the opposite poles of a dividing cell. This occurs because tubulin, their major constituent, has maintained a highly conserved amino acid sequence across all eukaryotic lineages, confining complexity, in multicellular organisms, to a variable number of tubulin genes and corresponding introns, these latter mostly present at conserved positions [2]. Certainly, introns are not involved in alternative splicing because there has never been any evidence for the occurrence of a processed tubulin protein that may have originated from it. While waiting for improvements in deciphering the functional role of tubulin introns, their genomic conservative position has served one of the simplest and most convenient, albeit unspoken, ways of effectively barcoding species, particularly plant species. In fact, by a simple Exon-Primed Intron Crossing reaction, without the need of any sequencing and any preliminary information on the genome of interest, a species–specific genomic profile can be obtained from any plant species so far investigated, now in the range of hundreds [7]. TBB, which is based on multiple nuclear intron-length polymorphisms, releases a true barcode, with no binnings, where the number and the size of the amplicons, resolved as peaks by capillary electrophoresis, are distinct and exclusive for any given plant species. In this way, it becomes very easy to build up a dedicated database that stores the genomic profiles of any plant species, including those most problematic such as herbals, ornamentals, and wild accessions. In fact, we found that trespassing the species boundary commonly associates to a radical change in the pattern of the TBB, for still unknown reasons. The TBB database is thus conveniently set up by recording the genomic profiles of any species or varieties of reference, each characterized by its specific number of peaks and related intron length polymorphisms, in the simple form of a spreadsheet file.

TBB also works well in mixtures where the presence of different species (up to 10) can be identified thanks to the presence of some specific, diagnostic peaks. This has been systematically verified and shown for the qualitative analysis of animal feed, typically made up by the combination of different raw materials of plant origin [8]. To this regard, TBB should actually be considered a metabarcoding technique because it can recognize a wide range of species in a single experiment. In addition, TBB can also be effective at the level of varieties, as shown in grapes and olives, producing data as good and consistent as those produced by internationally selected panels of multiple SSR markers. Varieties recognition does not hinder species discrimination because the changes in the genomic profile are far less numerous, and safe attribution can be made by storing correct reference samples in the dedicated database. Actually, TBB applied to grape genomic fingerprinting uncovered the presence of a new allele in Pinot Noir [9], missed by the *Vitis vinifera* genome sequencing project (*V. vinifera* GenomeDataBase; VvGDB, versionGenoscope12x). TBB works well at different taxonomical levels and allows an easy recognition of the parental contribution in hybrids, thus resolving a problem of identification that may come from different crossing events, spontaneous or planned [10]. TBB has also been shown to be successful in the identification of possible mistakes in the correct classification and registration of seed germplasm. Finally, alternative techniques such as oligonucleotide or bead-based arrays can be further applied to TBB-mediated species and varieties recognition [11,12].

Referring to its actual limits, TBB is currently successful in plants, is under implementation in animals but its application in fungi, or protists, is unlikely because of their limited number of beta-tubulin genes, often deprived of introns: An additional obstacle may be found when intron positioning is not conserved. In plants, TBB has been successfully tested in more than 150 species, mainly angiosperms, and the only problem occasionally found was when, as observed in orchids, the majority of DNA polymorphisms concentrate in introns longer than 1200 bp, the actual limit of resolution for capillary electrophoresis. In any case, a distinct genomic profile for any of the analyzed species has always been detected, but their number constitutes a small fraction of the total genetic resources. Nevertheless, the likelihood that a distinct TBB can be attributed at each plant species that contributes to the overall genetic diversity is high and deserves further verification because TBB offers a fast, largely applicable and valid alternative to any other form of classification based on genome sequencing.

Actually, massive sequencing technologies can be conveniently adapted to TBB by developing software that can rapidly and systematically analyze the multiple nucleotide sequences of the TBB amplicons, thus contributing to a conclusive step toward a full coverage of plant species and varieties discrimination and storage of information. Objections could be that the TBB system is all based on only one gene, but this feature is actually the key to success because, in comparison to *cox1* and any desirable plant equivalent gene, TBB bases its power of discrimination on multiple, essentially unlinked genes, belonging to the same gene family but distributed across different chromosomes. In conclusion, we think that the TBB story must be taken as paradigmatic for the preservation of hypothesis-driven experimental approaches and investigations that, despite the undoubted overwhelming power and undisputable contribution that may come by the massive technology focused on the BIG DATA, should not be abandoned. The two approaches should actually find a reciprocal strengthening and recognition as recently shown for the identification of the genetic determinants of the juvenile mortality in Braunvieh cattle, where the homozygous aplotype deficiency BH2 was eventually narrowed down to mutations occurring in the bovine *TUBD1* (tubulin Δ1) gene that causes a defect in microtubule organization of the respiratory cilia [13]. Applied to the barcoding of species, such a strategy could, at the very least, exploit TBB for supporting DNA barcoding sensu Hebert [3], possibly in association with DNA fingerprint methods based on the full sequencing of the mitochondrial or chloroplast genomes.
